# Bis(2-amino­benzothia­zol-3-ium) bis­(7-oxabicyclo­[2.2.1]heptane-2,3-dicarboxyl­ato-κ^3^ *O* ^2^,*O* ^3^,*O* ^7^)nickelate(II) hexa­hydrate

**DOI:** 10.1107/S1600536812017722

**Published:** 2012-04-28

**Authors:** Gui-Xian Wang, Qi-Wei Zhang, Fan Zhang

**Affiliations:** a Department of Chemistry, Lishui University, Lishui 323000, Zhejiang, People’s Republic of China; b College of Chemistry and Life Science, Zhejiang Normal University, Jinhua 321004, Zhejiang, People’s Republic of China

## Abstract

In the title compound, (C_7_H_7_N_2_S)_2_[Ni(C_8_H_8_O_5_)_2_]·6H_2_O, the Ni^II^ cation is located on an inversion center and is *O*,*O*′,*O*′′-chelated by two symmetry-related 7-oxabicyclo­[2.2.1]heptane-2,3-dicarboxyl­ate anions in a distorted octa­hedral geometry. The 2-amino­benzothia­zol-3-ium cation links with the Ni complex anion *via* N—H⋯O hydrogen bonding. Extensive O—H⋯O and N—H⋯O hydrogen bonds involving the lattice water mol­ecules also occur in the crystal structure.

## Related literature

For background to the applications of norcantharidin (systematic name: 7-oxabicyclo­[2,2,1]heptane-2,3-dicarb­oxy­lic anhydride), see: Hill *et al.* (2007 ▶). The isotypic Mn^II^, Co^II^ and Ni^II^ analogues were reported by Wang *et al.* (2010*a* ▶,*b* ▶) and Zhang *et al.* (2012 ▶), respectively.
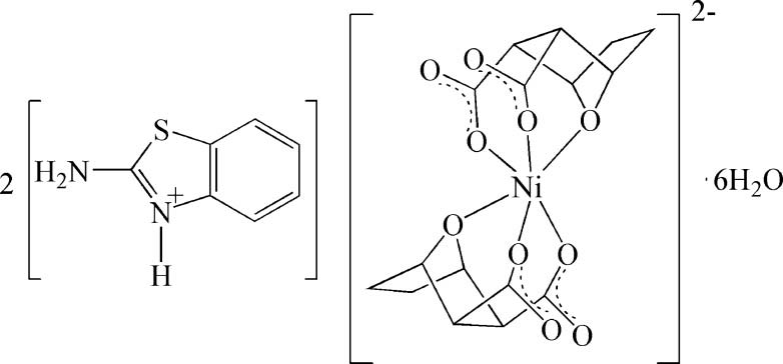


## Experimental

### 

#### Crystal data

(C_7_H_7_N_2_S)_2_[Ni(C_8_H_8_O_5_)_2_]·6H_2_O
*M* *_r_* = 837.51
Triclinic, 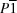

*a* = 6.6907 (1) Å
*b* = 10.0963 (2) Å
*c* = 13.2283 (3) Å
α = 90.284 (1)°
β = 91.192 (1)°
γ = 99.709 (1)°
*V* = 880.57 (3) Å^3^
*Z* = 1
Mo *K*α radiation
μ = 0.75 mm^−1^
*T* = 296 K
0.27 × 0.21 × 0.07 mm

#### Data collection

Bruker APEXII area-detector diffractometer
Absorption correction: multi-scan (*SADABS*; Sheldrick, 1996 ▶) *T*_min_ = 0.828, *T*_max_ = 0.951
11942 measured reflections
3083 independent reflections
2654 reflections with *I* > 2σ(*I*)
*R*_int_ = 0.032

#### Refinement

*R*[*F* ^2^ > 2σ(*F* ^2^)] = 0.032
*wR*(*F* ^2^) = 0.089
*S* = 1.06
3083 reflections
241 parameters
3 restraints
H-atom parameters constrained
Δρ_max_ = 0.38 e Å^−3^
Δρ_min_ = −0.44 e Å^−3^

### 

Data collection: *APEX2* (Bruker, 2006 ▶); cell refinement: *SAINT* (Bruker, 2006 ▶); data reduction: *SAINT*; program(s) used to solve structure: *SHELXS97* (Sheldrick, 2008 ▶); program(s) used to refine structure: *SHELXL97* (Sheldrick, 2008 ▶); molecular graphics: *SHELXTL* (Sheldrick, 2008 ▶); software used to prepare material for publication: *SHELXL97*.

## Supplementary Material

Crystal structure: contains datablock(s) I, global. DOI: 10.1107/S1600536812017722/xu5517sup1.cif

Structure factors: contains datablock(s) I. DOI: 10.1107/S1600536812017722/xu5517Isup2.hkl

Additional supplementary materials:  crystallographic information; 3D view; checkCIF report

## Figures and Tables

**Table 1:** Hydrogen-bond geometry (Å, °)

*D*—H⋯*A*	*D*—H	H⋯*A*	*D*⋯*A*	*D*—H⋯*A*
O1*W*—H1*WA*⋯O4^i^	0.85	1.97	2.815 (2)	179
O1*W*—H1*WB*⋯O3*W*	0.85	2.28	3.029 (3)	147
O2*W*—H2*WA*⋯O2^ii^	0.85	1.83	2.682 (2)	179
O2*W*—H2*WB*⋯O1*W*^iii^	0.85	1.95	2.798 (3)	178
O3*W*—H3*WA*⋯O1*W*^iv^	0.85	1.92	2.769 (3)	179
O3*W*—H3*WB*⋯O2*W*	0.85	1.95	2.772 (3)	161
N1—H1*A*⋯O4^i^	0.86	1.82	2.673 (2)	173
N2—H2*A*⋯O3^i^	0.86	2.01	2.863 (2)	173
N2—H2*B*⋯O2*W*^i^	0.86	2.00	2.818 (3)	158

Symmetry codes: (i) 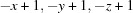; (ii) 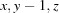; (iii) 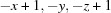; (iv) 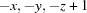.

**Fig. 1.**
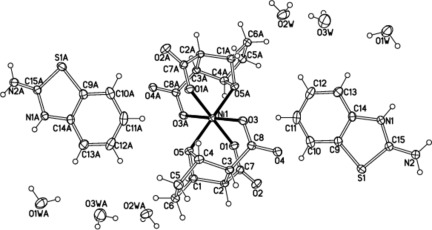
A view of the molecule of (I) showing the atom-labelling scheme with displacement ellipsoids drawn at the 30% probability.

## supplementary crystallographic information

### Comment

7-oxabicyclo[2,2,1]heptane-2,3-dicarboxylic anhydride (norcantharidin), which has been considered as potent inhibitor of the serine/threonine protein, has great anti-cancer activity (Hill *et al.*, 2007). A isostructural manganese complex (Wang *et al.*, 2010*a*) and a cobalt complex (Wang *et al.*, 2010*b*) has been reported. The molecular structure of the title complex is shown in Fig.1. The nickel atom is six-coordinated in a distorted octahedral coordination mode, binding to two bridging O atoms of the bicycloheptane unit and four carboxylate O atoms of two symmetry-related and fully deprotonated ligands. 2-aminobenzothiazole don't involved the coordination, and N atom of thiazole ring is protonated. The crystal structure is stabilized by N—H···O hydrogen-bonding interactions between the cations and anions and O—H···O hydrogen bonds including the crystal water molecules.

### Experimental

A mixture of 0.5 mmol norcantharidin, 0.5 mmol nickel acetate, 0.5 mmol 2-aminobenzothiazole and 15 mL distilled water was sealed in a 25 mL Teflon-lined stainless vessel and heated at 443 K for 3 d, then cooled slowly to room temperature. The solution was filtered and block green crystals were obtained.

### Refinement

The H atoms bonded to O atoms were located in a difference Fourier maps and refined with O—H distance restraints of 0.85 (3) Å and *U*_iso_(H) = 1.5*U*_eq_(O). Other H atoms were positioned geometrically and refined using a riding model with C—H = 0.97–0.98 and N—H = 0.86 Å, *U*_iso_(H) = 1.2*U*_eq_(C,N).

### Crystal data

**Table:**

(C_7_H_7_N_2_S)_2_[Ni(C_8_H_8_O_5_)_2_]·6H_2_O	*Z* = 1
*M**_r_* = 837.51	*F*(000) = 438
Triclinic, *P*1	*D*_x_ = 1.579 Mg m^−^^3^
Hall symbol: -P 1	Mo *K*α radiation, λ = 0.71073 Å
*a* = 6.6907 (1) Å	Cell parameters from 3486 reflections
*b* = 10.0963 (2) Å	θ = 1.5–25.0°
*c* = 13.2283 (3) Å	µ = 0.75 mm^−^^1^
α = 90.284 (1)°	*T* = 296 K
β = 91.192 (1)°	Block, green
γ = 99.709 (1)°	0.27 × 0.21 × 0.07 mm
*V* = 880.57 (3) Å^3^	

### Data collection

**Table:**

Bruker APEXII area-detector diffractometer	3083 independent reflections
Radiation source: fine-focus sealed tube	2654 reflections with *I* > 2σ(*I*)
Graphite monochromator	*R*_int_ = 0.032
ω scans	θ_max_ = 25.0°, θ_min_ = 1.5°
Absorption correction: multi-scan (*SADABS*; Sheldrick, 1996)	*h* = −7→7
*T*_min_ = 0.828, *T*_max_ = 0.951	*k* = −11→11
11942 measured reflections	*l* = −15→15

### Refinement

**Table:**

Refinement on *F*^2^	Primary atom site location: structure-invariant direct methods
Least-squares matrix: full	Secondary atom site location: difference Fourier map
*R*[*F*^2^ > 2σ(*F*^2^)] = 0.032	Hydrogen site location: inferred from neighbouring sites
*wR*(*F*^2^) = 0.089	H-atom parameters constrained
*S* = 1.06	*w* = 1/[σ^2^(*F*_o_^2^) + (0.0485*P*)^2^ + 0.2846*P*] where *P* = (*F*_o_^2^ + 2*F*_c_^2^)/3
3083 reflections	(Δ/σ)_max_ < 0.001
241 parameters	Δρ_max_ = 0.38 e Å^−^^3^
3 restraints	Δρ_min_ = −0.44 e Å^−^^3^

### Special details

**Table:**

Geometry. All e.s.d.'s (except the e.s.d. in the dihedral angle between two l.s. planes) are estimated using the full covariance matrix. The cell e.s.d.'s are taken into account individually in the estimation of e.s.d.'s in distances, angles and torsion angles; correlations between e.s.d.'s in cell parameters are only used when they are defined by crystal symmetry. An approximate (isotropic) treatment of cell e.s.d.'s is used for estimating e.s.d.'s involving l.s. planes.
Refinement. Refinement of *F*^2^ against ALL reflections. The weighted *R*-factor *wR* and goodness of fit *S* are based on *F*^2^, conventional *R*-factors *R* are based on *F*, with *F* set to zero for negative *F*^2^. The threshold expression of *F*^2^ > σ(*F*^2^) is used only for calculating *R*-factors(gt) *etc*. and is not relevant to the choice of reflections for refinement. *R*-factors based on *F*^2^ are statistically about twice as large as those based on *F*, and *R*- factors based on ALL data will be even larger.

### Fractional atomic coordinates and isotropic or equivalent isotropic displacement parameters (Å^2^)

**Table:**

	*x*	*y*	*z*	*U*_iso_*/*U*_eq_	
Ni1	0.5000	0.5000	0.0000	0.02482 (13)	
S1	0.32763 (10)	0.76888 (6)	0.52853 (4)	0.03601 (17)	
O1	0.3599 (2)	0.64286 (15)	0.06025 (12)	0.0324 (4)	
O1W	0.1930 (3)	0.1034 (2)	0.62809 (15)	0.0559 (5)	
H1WA	0.2029	0.1747	0.6636	0.084*	
H1WB	0.2102	0.1218	0.5659	0.084*	
H2WA	0.4613	−0.0379	0.2341	0.084*	
H2WB	0.6001	−0.0155	0.3078	0.084*	
H3WA	0.0734	−0.0017	0.3946	0.084*	
H3WB	0.2721	0.0206	0.3603	0.084*	
O2	0.3555 (2)	0.83685 (16)	0.13816 (12)	0.0374 (4)	
O2W	0.5108 (3)	0.02150 (17)	0.27766 (14)	0.0495 (5)	
O3	0.6790 (2)	0.51244 (15)	0.13147 (11)	0.0330 (4)	
O3W	0.1917 (3)	0.0438 (2)	0.40342 (16)	0.0651 (6)	
O4	0.7719 (3)	0.65778 (16)	0.25730 (11)	0.0358 (4)	
O5	0.7091 (2)	0.65098 (14)	−0.06734 (11)	0.0284 (3)	
N1	0.2773 (3)	0.53206 (18)	0.60097 (13)	0.0275 (4)	
H1A	0.2670	0.4669	0.6432	0.033*	
N2	0.3472 (3)	0.6994 (2)	0.72380 (14)	0.0369 (5)	
H2A	0.3401	0.6413	0.7715	0.044*	
H2B	0.3734	0.7838	0.7380	0.044*	
C6	0.8514 (4)	0.8692 (2)	−0.10948 (17)	0.0347 (5)	
H6A	0.8737	0.9613	−0.0848	0.042*	
H6B	0.8424	0.8689	−0.1828	0.042*	
C5	1.0189 (4)	0.7933 (2)	−0.07100 (18)	0.0356 (5)	
H5A	1.0851	0.7565	−0.1266	0.043*	
H5B	1.1199	0.8510	−0.0299	0.043*	
C1	0.6635 (3)	0.7873 (2)	−0.06430 (16)	0.0277 (5)	
H1B	0.5377	0.7965	−0.1009	0.033*	
C4	0.8995 (3)	0.6828 (2)	−0.00850 (16)	0.0286 (5)	
H4A	0.9683	0.6053	0.0013	0.034*	
C2	0.6554 (3)	0.8135 (2)	0.04989 (15)	0.0253 (5)	
H2C	0.6916	0.9099	0.0643	0.030*	
C3	0.8288 (3)	0.7379 (2)	0.08999 (16)	0.0259 (5)	
H3A	0.9391	0.8026	0.1208	0.031*	
C7	0.4422 (3)	0.7606 (2)	0.08724 (16)	0.0271 (5)	
C8	0.7543 (3)	0.6281 (2)	0.16535 (16)	0.0261 (5)	
C9	0.2752 (3)	0.6322 (2)	0.44420 (17)	0.0305 (5)	
C10	0.2573 (4)	0.6336 (3)	0.34006 (18)	0.0417 (6)	
H10A	0.2723	0.7142	0.3050	0.050*	
C11	0.2165 (4)	0.5119 (3)	0.28954 (19)	0.0466 (7)	
H11A	0.2056	0.5102	0.2193	0.056*	
C12	0.1917 (4)	0.3922 (3)	0.34191 (19)	0.0433 (6)	
H12A	0.1622	0.3114	0.3062	0.052*	
C13	0.2098 (3)	0.3900 (3)	0.44640 (18)	0.0356 (5)	
H13A	0.1941	0.3093	0.4813	0.043*	
C14	0.2521 (3)	0.5118 (2)	0.49681 (16)	0.0278 (5)	
C15	0.3181 (3)	0.6592 (2)	0.62951 (16)	0.0284 (5)	

### Atomic displacement parameters (Å^2^)

**Table:**

	*U*^11^	*U*^22^	*U*^33^	*U*^12^	*U*^13^	*U*^23^
Ni1	0.0312 (2)	0.0187 (2)	0.0230 (2)	0.00043 (16)	−0.00167 (16)	−0.00208 (15)
S1	0.0480 (4)	0.0277 (3)	0.0308 (3)	0.0020 (3)	−0.0013 (3)	0.0062 (2)
O1	0.0328 (8)	0.0246 (8)	0.0385 (9)	0.0009 (7)	0.0025 (7)	−0.0048 (7)
O1W	0.0682 (13)	0.0494 (12)	0.0491 (11)	0.0083 (10)	−0.0104 (10)	−0.0136 (9)
O2	0.0374 (9)	0.0340 (9)	0.0420 (9)	0.0097 (7)	0.0030 (8)	−0.0128 (7)
O2W	0.0574 (12)	0.0350 (10)	0.0550 (11)	0.0068 (9)	−0.0154 (9)	−0.0122 (8)
O3	0.0443 (9)	0.0234 (8)	0.0281 (8)	−0.0023 (7)	−0.0070 (7)	0.0032 (6)
O3W	0.0550 (12)	0.0735 (15)	0.0625 (13)	−0.0023 (11)	0.0085 (10)	−0.0073 (11)
O4	0.0520 (10)	0.0285 (9)	0.0250 (8)	0.0017 (7)	−0.0048 (7)	0.0019 (6)
O5	0.0357 (8)	0.0213 (8)	0.0266 (8)	0.0006 (6)	−0.0005 (7)	−0.0037 (6)
N1	0.0330 (10)	0.0266 (10)	0.0230 (9)	0.0049 (8)	−0.0001 (8)	0.0033 (7)
N2	0.0556 (13)	0.0273 (10)	0.0265 (10)	0.0036 (9)	−0.0016 (9)	0.0004 (8)
C6	0.0505 (15)	0.0250 (12)	0.0266 (11)	0.0000 (10)	0.0037 (11)	0.0014 (9)
C5	0.0372 (13)	0.0330 (13)	0.0352 (13)	0.0012 (10)	0.0068 (11)	−0.0012 (10)
C1	0.0339 (12)	0.0226 (11)	0.0264 (11)	0.0051 (9)	−0.0043 (9)	0.0014 (9)
C4	0.0284 (11)	0.0247 (11)	0.0328 (12)	0.0051 (9)	−0.0005 (10)	−0.0011 (9)
C2	0.0317 (12)	0.0176 (10)	0.0256 (11)	0.0021 (9)	−0.0015 (9)	−0.0012 (8)
C3	0.0284 (11)	0.0209 (11)	0.0263 (11)	−0.0014 (9)	−0.0034 (9)	−0.0013 (9)
C7	0.0331 (12)	0.0251 (12)	0.0235 (10)	0.0062 (9)	−0.0029 (9)	−0.0015 (9)
C8	0.0260 (11)	0.0251 (11)	0.0261 (11)	0.0019 (9)	−0.0059 (9)	0.0025 (9)
C9	0.0291 (12)	0.0351 (13)	0.0267 (11)	0.0039 (10)	0.0005 (9)	0.0016 (9)
C10	0.0430 (14)	0.0522 (16)	0.0298 (12)	0.0069 (12)	0.0026 (11)	0.0089 (12)
C11	0.0444 (15)	0.071 (2)	0.0244 (12)	0.0112 (14)	0.0016 (11)	−0.0049 (13)
C12	0.0362 (14)	0.0556 (17)	0.0378 (14)	0.0075 (12)	−0.0013 (11)	−0.0194 (12)
C13	0.0319 (12)	0.0374 (14)	0.0379 (13)	0.0067 (10)	0.0014 (10)	−0.0053 (11)
C14	0.0230 (11)	0.0353 (13)	0.0253 (11)	0.0051 (9)	0.0023 (9)	0.0016 (9)
C15	0.0298 (12)	0.0292 (12)	0.0262 (11)	0.0049 (9)	0.0006 (9)	0.0024 (9)

### Geometric parameters (Å, º)

**Table:**

Ni1—O1^i^	2.0174 (15)	C6—C1	1.520 (3)
Ni1—O1	2.0174 (15)	C6—C5	1.541 (3)
Ni1—O3^i^	2.0823 (15)	C6—H6A	0.9700
Ni1—O3	2.0823 (15)	C6—H6B	0.9700
Ni1—O5	2.1024 (14)	C5—C4	1.517 (3)
Ni1—O5^i^	2.1024 (14)	C5—H5A	0.9700
S1—C15	1.735 (2)	C5—H5B	0.9700
S1—C9	1.754 (2)	C1—C2	1.536 (3)
O1—C7	1.269 (3)	C1—H1B	0.9800
O1W—H1WA	0.8500	C4—C3	1.529 (3)
O1W—H1WB	0.8500	C4—H4A	0.9800
O2—C7	1.243 (3)	C2—C7	1.529 (3)
O2W—H2WA	0.8502	C2—C3	1.577 (3)
O2W—H2WB	0.8503	C2—H2C	0.9800
O3—C8	1.267 (3)	C3—C8	1.522 (3)
O3W—H3WA	0.8503	C3—H3A	0.9800
O3W—H3WB	0.8503	C9—C10	1.381 (3)
O4—C8	1.250 (3)	C9—C14	1.391 (3)
O5—C1	1.459 (3)	C10—C11	1.379 (4)
O5—C4	1.467 (3)	C10—H10A	0.9300
N1—C15	1.318 (3)	C11—C12	1.383 (4)
N1—C14	1.394 (3)	C11—H11A	0.9300
N1—H1A	0.8600	C12—C13	1.386 (3)
N2—C15	1.310 (3)	C12—H12A	0.9300
N2—H2A	0.8600	C13—C14	1.380 (3)
N2—H2B	0.8600	C13—H13A	0.9300
			
O1^i^—Ni1—O1	180.0	C2—C1—H1B	113.3
O1^i^—Ni1—O3^i^	87.63 (6)	O5—C4—C5	101.81 (17)
O1—Ni1—O3^i^	92.37 (6)	O5—C4—C3	101.95 (16)
O1^i^—Ni1—O3	92.37 (6)	C5—C4—C3	111.62 (18)
O1—Ni1—O3	87.63 (6)	O5—C4—H4A	113.4
O3^i^—Ni1—O3	180.00 (9)	C5—C4—H4A	113.4
O1^i^—Ni1—O5	90.51 (6)	C3—C4—H4A	113.4
O1—Ni1—O5	89.49 (6)	C7—C2—C1	109.66 (17)
O3^i^—Ni1—O5	89.20 (6)	C7—C2—C3	116.01 (17)
O3—Ni1—O5	90.80 (6)	C1—C2—C3	100.63 (16)
O1^i^—Ni1—O5^i^	89.49 (6)	C7—C2—H2C	110.0
O1—Ni1—O5^i^	90.51 (6)	C1—C2—H2C	110.0
O3^i^—Ni1—O5^i^	90.80 (6)	C3—C2—H2C	110.0
O3—Ni1—O5^i^	89.20 (6)	C8—C3—C4	112.85 (17)
O5—Ni1—O5^i^	180.0	C8—C3—C2	112.99 (17)
C15—S1—C9	90.16 (11)	C4—C3—C2	101.37 (16)
C7—O1—Ni1	126.63 (14)	C8—C3—H3A	109.8
H1WA—O1W—H1WB	111.0	C4—C3—H3A	109.8
H2WA—O2W—H2WB	102.3	C2—C3—H3A	109.8
C8—O3—Ni1	118.10 (13)	O2—C7—O1	123.6 (2)
H3WA—O3W—H3WB	110.3	O2—C7—C2	118.55 (19)
C1—O5—C4	95.27 (15)	O1—C7—C2	117.79 (18)
C1—O5—Ni1	116.89 (12)	O4—C8—O3	123.94 (19)
C4—O5—Ni1	112.33 (12)	O4—C8—C3	117.72 (19)
C15—N1—C14	114.58 (18)	O3—C8—C3	118.34 (18)
C15—N1—H1A	122.7	C10—C9—C14	121.1 (2)
C14—N1—H1A	122.7	C10—C9—S1	128.6 (2)
C15—N2—H2A	120.0	C14—C9—S1	110.31 (16)
C15—N2—H2B	120.0	C11—C10—C9	118.0 (2)
H2A—N2—H2B	120.0	C11—C10—H10A	121.0
C1—C6—C5	101.64 (17)	C9—C10—H10A	121.0
C1—C6—H6A	111.4	C10—C11—C12	120.9 (2)
C5—C6—H6A	111.4	C10—C11—H11A	119.6
C1—C6—H6B	111.4	C12—C11—H11A	119.6
C5—C6—H6B	111.4	C11—C12—C13	121.4 (2)
H6A—C6—H6B	109.3	C11—C12—H12A	119.3
C4—C5—C6	101.95 (19)	C13—C12—H12A	119.3
C4—C5—H5A	111.4	C14—C13—C12	117.6 (2)
C6—C5—H5A	111.4	C14—C13—H13A	121.2
C4—C5—H5B	111.4	C12—C13—H13A	121.2
C6—C5—H5B	111.4	C13—C14—C9	120.9 (2)
H5A—C5—H5B	109.2	C13—C14—N1	126.8 (2)
O5—C1—C6	102.53 (17)	C9—C14—N1	112.23 (19)
O5—C1—C2	102.03 (16)	N2—C15—N1	124.1 (2)
C6—C1—C2	111.26 (18)	N2—C15—S1	123.20 (17)
O5—C1—H1B	113.3	N1—C15—S1	112.73 (16)
C6—C1—H1B	113.3		
			
O3^i^—Ni1—O1—C7	−120.43 (17)	C5—C4—C3—C2	72.7 (2)
O3—Ni1—O1—C7	59.57 (17)	C7—C2—C3—C8	−3.4 (2)
O5—Ni1—O1—C7	−31.25 (17)	C1—C2—C3—C8	−121.57 (18)
O5^i^—Ni1—O1—C7	148.75 (17)	C7—C2—C3—C4	117.67 (19)
O1^i^—Ni1—O3—C8	134.35 (16)	C1—C2—C3—C4	−0.53 (19)
O1—Ni1—O3—C8	−45.65 (16)	Ni1—O1—C7—O2	−168.26 (16)
O5—Ni1—O3—C8	43.80 (16)	Ni1—O1—C7—C2	15.2 (3)
O5^i^—Ni1—O3—C8	−136.20 (16)	C1—C2—C7—O2	−128.9 (2)
O1^i^—Ni1—O5—C1	169.62 (13)	C3—C2—C7—O2	118.0 (2)
O1—Ni1—O5—C1	−10.38 (13)	C1—C2—C7—O1	47.8 (2)
O3^i^—Ni1—O5—C1	82.01 (13)	C3—C2—C7—O1	−65.2 (2)
O3—Ni1—O5—C1	−97.99 (13)	Ni1—O3—C8—O4	142.42 (18)
O1^i^—Ni1—O5—C4	−81.74 (13)	Ni1—O3—C8—C3	−37.8 (2)
O1—Ni1—O5—C4	98.26 (13)	C4—C3—C8—O4	151.24 (19)
O3^i^—Ni1—O5—C4	−169.35 (13)	C2—C3—C8—O4	−94.5 (2)
O3—Ni1—O5—C4	10.65 (13)	C4—C3—C8—O3	−28.5 (3)
C1—C6—C5—C4	−1.2 (2)	C2—C3—C8—O3	85.8 (2)
C4—O5—C1—C6	56.54 (18)	C15—S1—C9—C10	−179.3 (2)
Ni1—O5—C1—C6	174.86 (12)	C15—S1—C9—C14	0.40 (17)
C4—O5—C1—C2	−58.75 (18)	C14—C9—C10—C11	−0.2 (4)
Ni1—O5—C1—C2	59.58 (17)	S1—C9—C10—C11	179.49 (19)
C5—C6—C1—O5	−34.3 (2)	C9—C10—C11—C12	0.8 (4)
C5—C6—C1—C2	74.1 (2)	C10—C11—C12—C13	−1.0 (4)
C1—O5—C4—C5	−57.14 (18)	C11—C12—C13—C14	0.5 (4)
Ni1—O5—C4—C5	−179.07 (13)	C12—C13—C14—C9	0.1 (3)
C1—O5—C4—C3	58.27 (18)	C12—C13—C14—N1	−179.7 (2)
Ni1—O5—C4—C3	−63.67 (16)	C10—C9—C14—C13	−0.2 (3)
C6—C5—C4—O5	36.1 (2)	S1—C9—C14—C13	180.00 (18)
C6—C5—C4—C3	−72.0 (2)	C10—C9—C14—N1	179.5 (2)
O5—C1—C2—C7	−86.34 (19)	S1—C9—C14—N1	−0.2 (2)
C6—C1—C2—C7	164.94 (18)	C15—N1—C14—C13	179.6 (2)
O5—C1—C2—C3	36.40 (19)	C15—N1—C14—C9	−0.2 (3)
C6—C1—C2—C3	−72.3 (2)	C14—N1—C15—N2	−179.9 (2)
O5—C4—C3—C8	85.9 (2)	C14—N1—C15—S1	0.5 (2)
C5—C4—C3—C8	−166.13 (18)	C9—S1—C15—N2	179.9 (2)
O5—C4—C3—C2	−35.25 (19)	C9—S1—C15—N1	−0.52 (18)

Symmetry code: (i) −*x*+1, −*y*+1, −*z*.

### Hydrogen-bond geometry (Å, º)

**Table:**

*D*—H···*A*	*D*—H	H···*A*	*D*···*A*	*D*—H···*A*
O1*W*—H1*WA*···O4^ii^	0.85	1.97	2.815 (2)	179
O1*W*—H1*WB*···O3*W*	0.85	2.28	3.029 (3)	147
O2*W*—H2*WA*···O2^iii^	0.85	1.83	2.682 (2)	179
O2*W*—H2*WB*···O1*W*^iv^	0.85	1.95	2.798 (3)	178
O3*W*—H3*WA*···O1*W*^v^	0.85	1.92	2.769 (3)	179
O3*W*—H3*WB*···O2*W*	0.85	1.95	2.772 (3)	161
N1—H1*A*···O4^ii^	0.86	1.82	2.673 (2)	173
N2—H2*A*···O3^ii^	0.86	2.01	2.863 (2)	173
N2—H2*B*···O2*W*^ii^	0.86	2.00	2.818 (3)	158

Symmetry codes: (ii) −*x*+1, −*y*+1, −*z*+1; (iii) *x*, *y*−1, *z*; (iv) −*x*+1, −*y*, −*z*+1; (v) −*x*, −*y*, −*z*+1.

## References

[bb1] Bruker (2006). *APEX2* and *SAINT* Bruker AXS Inc., Madison, Wisconsin, USA.

[bb2] Hill, T.-A., Stewart, S.-G., Sauer, B., Gilbert, J., Ackland, S.-P., Sakoff, J.-A. & McCluskey, A. (2007). *Bioorg. Med. Chem. Lett.* **17**, 3392–3397. 10.1016/j.bmcl.2007.03.093 17451951

[bb3] Sheldrick, G. M. (1996). *SADABS* University of Göttingen, Germany.

[bb4] Sheldrick, G. M. (2008). *Acta Cryst.* A**64**, 112–122. 10.1107/S0108767307043930 18156677

[bb5] Wang, N., Lin, Q.-Y., Feng, J., Li, S.-K. & Zhao, J.-J. (2010*b*). *Acta Cryst.* E**66**, m763–m764. 10.1107/S1600536810020921 PMC3006894 21587697

[bb6] Wang, N., Wen, Y.-H., Lin, Q.-Y. & Feng, J. (2010*a*). *Acta Cryst.* E**66**, m762. 10.1107/S160053681002091X PMC3007082 21587696

[bb7] Zhang, F., Lv, T.-X., Feng, J. & Lin, Q. Y. (2012). *Acta Cryst.* E**68**, m684. 10.1107/S1600536812017886 PMC3344404 22590166

